# A Retrospective Analysis of Medical Management Strategies for Trigeminal Neuralgia: An Institutional Review

**DOI:** 10.7759/cureus.69258

**Published:** 2024-09-12

**Authors:** Induja Murugesan, Umamaheswari T.N., Karthikeyan Ramalingam

**Affiliations:** 1 Oral Medicine and Radiology, Saveetha Dental College and Hospitals, Saveetha Institute of Medical and Technical Sciences, Saveetha University, Chennai, IND; 2 Oral Pathology and Microbiology, Saveetha Dental College and Hospitals, Saveetha Institute of Medical and Technical Sciences, Saveetha University, Chennai, IND

**Keywords:** carbamazepine (cbz), clinical predictors, combination therapy, gabapentin neuro, maintenance therapy, neuropathic pain, pain management, pain relief, pharmacotherapy, trigeminal neuralgia

## Abstract

Background

Neuropathic pain results from nervous system damage, and trigeminal neuralgia (TN), also called tic douloureux, is a chronic disorder affecting the trigeminal nerve. TN causes sudden, severe, recurring facial pain that can be highly disabling. Treatment usually involves medications like carbamazepine, oxcarbazepine, gabapentin, or baclofen to manage nerve pain. If medications are ineffective, surgical interventions like microvascular decompression or gamma knife radiosurgery may be explored for symptom relief.

Aim and objectives

This study aims to assess the demographic characteristics and clinical features of patients with TN treated at a private institution. The objective was to assess key demographic factors, including age, gender, and the affected trigeminal nerve divisions, with a focus on identifying the most frequently involved nerve divisions in TN cases.

Methodology

This five-year retrospective study (January 2019-April 2023) in the Oral Medicine and Radiology Department analyzed 483 TN cases, including 300 patients with confirmed primary TN and complete records. Dental Information Archive System (DIAS) data covered demographics, clinical features, treatments, and outcomes. Pain levels were assessed using the visual analog scale (VAS). LFTs monitor the long-term effects of medications like carbamazepine. Statistical analysis employed descriptive statistics, chi-square tests, and t-tests, with p < 0.05 considered significant.

Results

Of 7,500 outpatients in the Oral Medicine Department, 483 were diagnosed with TN, and 300 met the diagnostic criteria for inclusion in a five-year study (January 2019-April 2023). The average age was 60 years for men and 58.5 years for women. TN primarily affected the right side in 158 patients (56%), while the left side was involved in 114 (43%) of the cases (p = 0.04) and most commonly involved the maxillary nerve (V2) in 159 patients (53%) and the mandibular nerve (V3) in 141 patients (47%), with a slight female predominance (p = 0.02). One hundred thirty-five patients (45%) used carbamazepine alone, while 84 were treated with carbamazepine and gabapentin, and 81 were treated with carbamazepine and baclofen. The combination of carbamazepine and gabapentin was the most effective, achieving pain control in 123 patients (75%) compared to 94 patients (70%) with carbamazepine alone and 119 patients (72%) with carbamazepine and baclofen (p = 0.06). VAS scores showed better pain relief with carbamazepine and gabapentin (VAS: 3.8 ± 1.0), carbamazepine alone (mean VAS: 4.5 ± 1.2) and carbamazepine with baclofen (mean VAS: 4.2 ± 1.1). In long-term management, 45 patients (15%) discontinued due to side effects, while 105 patients (35%) continued on carbamazepine alone, 90 patients (30%) on carbamazepine with gabapentin, and 60 patients (20%) on carbamazepine with baclofen (p = 0.04). LFTs were performed on 240 patients (80%), while 60 patients (20%) did not undergo LFTs.

Conclusions

This study underscores the treatment of TN, with anticonvulsants as the primary therapy and alternative options available for refractory cases. However, limitations like small sample size and lack of long-term follow-up affect the findings' generalizability. The results highlight the importance of treatment plans and the potential advantages of combination therapies in clinical practice.

## Introduction

The International Association for Study of Pain (IASP) in 2020 defined pain as “unpleasant sensory and emotional experience associated with or resembling that associated with actual or potential tissue damage” [1]. The International Headache Society (IHS) defines primary trigeminal neuralgia (TN) as "a unilateral disorder characterized by brief electric shock-like pains, abrupt in onset and termination, limited to the distribution of one or more divisions of the trigeminal nerve" [2]. The pain is severe and an electric shock, or as sharp, stabbing, or shooting in nature. It is triggered by a gentle touch and activities like eating or speaking [3]. The annual incidence is about 4.5 to 12.6 per 100,000 more in females than males [4]. Studies based on population data estimate that the lifetime frequency of TN ranges from 0.16% to 0.3% [5]. There is no strong epidemiology data available for India. The pain was more frequently reported in the maxillary (V2) division, followed by mandibular (V3) divisions and the ophthalmic (V1) division of the fifth cranial nerve [6]. The V2 and V3 divisions are more affected on the right-sided face than the left side [7]. TN affects the right side of the face more often, possibly due to narrower foramina leading to nerve entrapment. This asymmetry may play a key role in the higher incidence of right-sided TN [8].

The cause of TN is unknown. The pathogenesis of TN refers to the underlying causes and mechanisms that lead to the development of this condition. The most widely accepted theory is the ignition hypothesis by Wegal and Cassay, which suggests that blood vessel compression of the trigeminal nerve, typically at the root entry zone, leads to myelin sheath loss. This results in abnormal impulse transmission, known as ephaptic transmission, causing severe pain. About 95% of cases are due to this vessel-nerve interaction, termed primary or classical TN, while the remaining 5% are attributed to organic lesions such as tumors, aneurysms, infections, or autoimmune diseases, classified as secondary TN [9]. Clinically, TN has been described in terminologies like typical or classical TN and secondary TN [10]. The IHS diagnostic criteria for the diagnosis of classical TN are as follows: (a) paroxysmal attacks of pain lasting from a fraction of a second to two minutes, affecting one or more divisions of the trigeminal nerve; (b) pain has at least one of the following characteristics: (i) intense, sharp, superficial, or stabbing and (ii) precipitated from trigger areas or by trigger factors; (c) attacks are stereotyped in the individual; (d) there is no clinically evident neurological deficit; and (e) not attributed to another disorder. [11]

Various imaging modalities have been used to evaluate cranial nerves, with the trigeminal nerve being better visualized due to its size. Computed tomography offers good visibility of foramina and nerve exit points. Magnetic resonance imaging (MRI), especially with the right sequences, is the preferred method for cranial nerve imaging. Advances in MRI are crucial for presurgical evaluation of the trigeminal nerve. It has been employed as a therapeutic planning tool for trigeminal neural diseases. Short tau inversion recovery (STIR) in the coronal plane and T1-weighted spin echo sequences in the axial plane comprise a fundamental standardized method for patients exhibiting trigeminal nerve complaints [12]. The medical management of TN is carbamazepine and ox-carbamazepine. Carbamazepine is the gold standard treatment for primary TN should involve a daily dosage of 200-1,200 mg of carbamazepine and 600-1,800 mg of oxcarbazepine. The second-line treatment consists of three medications: gabapentin, lamotrigine, baclofen, and pimozide, and they are recommended for reducing pain in patients with classic TN [13]. The more recently tested antiepileptic drugs (AEDs) include gabapentin, pregabalin, topiramate, and levetiracetam [14]. For patients who did not achieve satisfactory relief through pharmacotherapy or who experienced significant side effects, interventional and surgical options were considered. This review revealed that many patients benefitted from procedures such as microvascular decompression (MVD), radiofrequency rhizotomy, or gamma knife radiosurgery, which are established as effective alternatives when conservative medical management fails [15].

This study aims to evaluate TN's demographic and clinical features in private institutions. To investigate the effectiveness of various medical management strategies for TN in private healthcare establishments and the various medicinal approaches employed to treat the condition. To improve the medical management strategies for TN, addressing varied patient responses and exploring alternatives to optimize treatment outcomes.

## Materials and methods

This retrospective observational study was conducted at a private institution's Oral Medicine and Radiology Department to evaluate patients' demographic and clinical features with TN and various treatment regimens. The five-year study period from January 2019 to April 2023 was selected to encompass recent advancements in diagnostic imaging and treatment approaches for TN, providing a thorough analysis of contemporary clinical practices. This study received approval from the Institutional Human Ethical Committee in the Saveetha Dental College and Hospital under ethical committee reference number IHEC/SDC/OMED-2105/23/144. A digitally signed consent form is available in the DIAS. The Research and Ethics Department, with our senior faculty, ensured that data privacy was maintained during the research phase.

The study population included 7,500 outpatients treated at the Oral Medicine and Radiology Department, among which 483 patients were diagnosed with TN based on clinical evaluation of sweet diagnostic criteria and, where applicable, diagnostic imaging like MRI. Among these, 300 patients were included and were selected for this study. The inclusion criteria required a confirmed TN diagnosis based on clinical criteria, including sudden, unilateral, electric shock-like facial pain typically triggered by routine activities (e.g., chewing or talking). Complete medical records, treatment with at least one standard pharmacological regimen (e.g., carbamazepine, carbamazepine with gabapentin, or carbamazepine with baclofen), and follow-up data to assess outcomes were also required. MRI was used to rule out structural causes such as tumors or vascular malformations. Patients with incomplete medical records or secondary TN causes (e.g., multiple sclerosis, tumors) were excluded to ensure the study focused solely on primary TN cases.

Records were selected based on clear documentation of diagnosis, treatment regimen, and follow-up outcomes. To ensure accuracy, data were verified by cross-checking information such as clinical notes, diagnostic imaging, and treatment histories. Incomplete data were handled by excluding records with missing key information (e.g., absence of treatment details or follow-up results) to maintain the integrity and reliability of the study’s findings. The VAS was used as the primary outcome measure to assess patient pain levels. The VAS scores, typically recorded in the patient's medical records during clinical visits, ranged from 0 (no pain) to 10 (worst possible pain), providing a subjective measurement of pain intensity before and after treatment. Other outcome measures, such as treatment response and adverse effects, were gathered from clinical notes and follow-up reports.

Patient data were collected through the Dental Information Archive System (DIAS). This comprehensive digital health records system facilitated the retrieval and review of medical histories, treatment details, and follow-up information. Key data points extracted from patient records included demographic information such as age, gender, and the laterality of TN, whether on the right or left side. Clinical features were documented, focusing on which trigeminal nerves were affected (V1, V2, or V3) and the specific symptomatology experienced. Treatment details encompassed the types of pharmacological therapy received, including carbamazepine alone or in combination with gabapentin or baclofen, along with dosage information and the duration of therapy. Pain levels were assessed using the VAS, with scores recorded both before and after treatment to gauge the effectiveness of the interventions. Additionally, blood investigations, particularly liver function tests (LFTs), were performed to monitor the long-term effects of medications, especially carbamazepine, and to ensure patient safety throughout treatment.

Descriptive statistics were used to summarize the demographic and clinical characteristics of the study population. Treatment effectiveness was assessed by comparing the proportion of patients achieving pain control, with results stratified by treatment type. Mean VAS scores and standard deviations were calculated for each treatment group. Parametric tests, such as t-tests, were chosen due to the normal distribution of continuous variables and sufficient sample sizes, allowing comparison of mean pain scores and categorical outcomes across treatment types. Statistical significance was evaluated using chi-square and t-tests, with a p-value of less than 0.05 considered significant.

## Results

**Figure 1:**
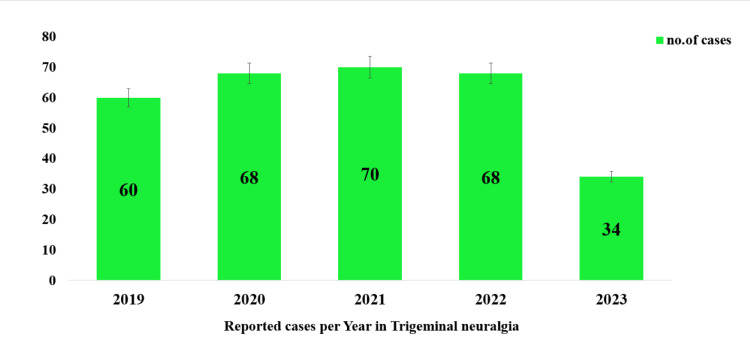
Number of reported cases from January 2019 to April 2023 in trigeminal neuralgia patients.

Out of the 7,500 outpatients in the Oral Medicine and Radiology Department, 483 were diagnosed with TN, which represents 6.44% of the total outpatients. Of these, 300 patients (62.11% of those diagnosed with TN) met the diagnostic criteria and were included in the study. Data were collected using the DIAS over five years, from January 2019 to April 2023, covering all 483 cases. The annual breakdown of TN cases included 60 in 2019, 68 in 2020, 70 in 2021, 68 in 2022, and 34 in 2023 (Figure 1).

The ages of male patients ranged from 38 to 87, with a mean of 60. The ages of female patients ranged from 35 to 82, with a mean of 58.5 (Table 1).

**Table 1:** Patients' mean age and gender

Variables	Males	Females	Total
No. of patients	140 (46.6%)	160 (53.3%)	300 (100%)
Mean age (years)	60.0 ± 7.5	58.5 ± 7	59.2 ± 7.3

**Figure 2:**
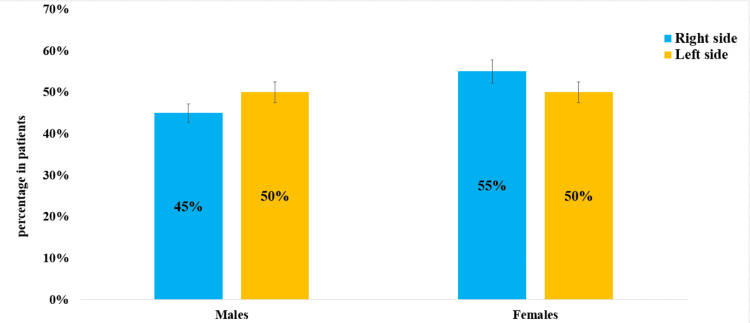
In this study population, the sides were impacted by trigeminal neuralgia. In this study, TN affected the right side in 98 females (55%) and 88 males (45%), while the left side was involved in 114 patients (43%), equally distributed with 57 females (50%) and 57 males (50%).

The study found that TN affected the right side and was involved in 158 (56%) patients of the study population, while the left side was involved in 114 (43%) of the cases. On the right side, TN affected 98 females, representing 55% of the right-sided cases, and 88 males, representing 45%. On the left side, the condition was equally distributed, with 57 females and 57 males, each making up 50% of the left sided. The right side is affected more frequently than the left side, with a significant value of (p = 0.04). The female predominance may suggest a potential hormonal or genetic influence, further exploration into how gender-specific factors contribute to TN susceptibility. The right-side involvement is particularly intriguing, as most cranial neuropathies typically do not show a clear lateral preference (Figure 2).

**Figure 3:**
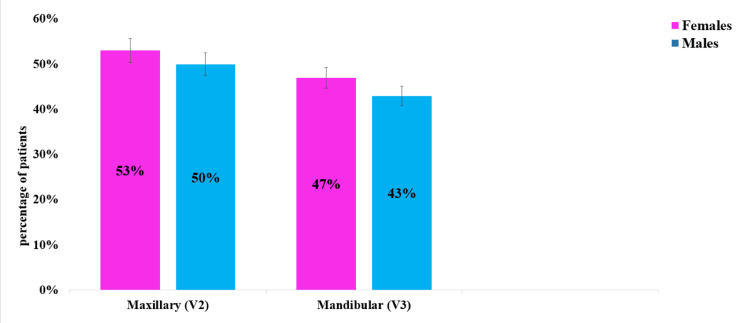
Frequency of involvement in trigeminal nerve branch in this study population. In our study, the maxillary nerve was affected in 159 patients (53%), with 81 females (53%) and 78 males (50%), while the mandibular nerve was involved in 141 patients (47%), with 78 females (47%) and 63 males (43%).

In our study, the maxillary nerve was affected in 159 patients, accounting for 53% of the cases, while the mandibular nerve was involved in 141 patients, representing 47% of the cases. The data revealed a slightly higher involvement in females compared to males across both nerve divisions. Specifically, in the maxillary division, TN affected 81 females (53%) and 78 males (50%). Similarly, in the mandibular division, TN was observed in 78 females (47%) and 63 males (43%). The maxillary branch was more frequently affected than the mandibular branch, with this difference being statistically significant (p = 0.02) (Figure 3).

Out of the 300 patients, 135 (45%) were treated with carbamazepine alone, starting with a dosage of 200 mg taken twice or thrice a day. The remaining 165 patients (55%) received combination therapy, with 84 (28%) treated with carbamazepine and gabapentin and 81 (27%) treated with carbamazepine and baclofen.

**Figure 4:**
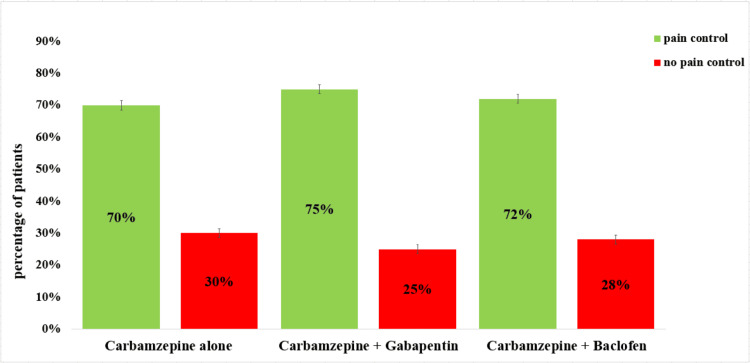
Pain control and no pain control in carbamazepine vs. carbamazepine with gabapentin vs. carbamazepine with baclofen. The graph shows that 94 (70%) of patients treated with carbamazepine alone experienced pain control, while 41 (30%) showed no pain control. The combination of carbamazepine and gabapentin 63 (75%) of patients reported pain control, and 21 (25%) showed no pain control. The combination of carbamazepine and baclofen resulted in 58 (72%) of patients achieving pain control, while 23 (28%) showed no pain control.

The comparative effectiveness of different pharmacological regimens in managing TN, focusing on pain control vs. no pain control among patients. Three treatment groups are presented: carbamazepine alone, carbamazepine combined with gabapentin, and carbamazepine combined with baclofen. The data show that 94 (70%) of patients treated with carbamazepine alone experienced pain control, while 41 (30%) did not achieve adequate relief. The combination of carbamazepine and gabapentin demonstrated the highest effectiveness, with 63 (75%) of patients reporting pain control and 21 (25%) showing no pain control. Similarly, the combination of carbamazepine and baclofen resulted in 58 (72%) of patients achieving pain control, while 23 (28%) did not experience sufficient relief, with a significant value (p = 0.06) (Figure 4).

In this study, the pain levels of patients with TN were assessed using the VAS before and after treatment across three groups: carbamazepine alone, carbamazepine combined with gabapentin, and carbamazepine combined with baclofen. The group treated with carbamazepine alone showed moderate pain control, with a mean VAS score of 4.5 after treatment. Patients receiving the combination of carbamazepine and gabapentin experienced better pain relief, with a mean VAS score of 3.8. Meanwhile, the group treated with carbamazepine and baclofen had a mean post-treatment VAS score of 4.2. These results suggest that combination therapies, particularly with gabapentin, provided more effective pain reduction compared to carbamazepine alone (Table 2).

**Table 2:** The mean and SD of VAS scores for the three treatment groups: carbamazepine alone, carbamazepine with gabapentin, and carbamazepine with baclofen SD, standard deviation; VAS, visual analog scale

Treatment group	Mean ± SD, VAS score
Carbamazepine alone	4.5 ± 1.2
Carbamazepine with gabapentin	3.8 ± 1.0
Carbamazepine with baclofen	4.2 ± 1.1

**Figure 5:**
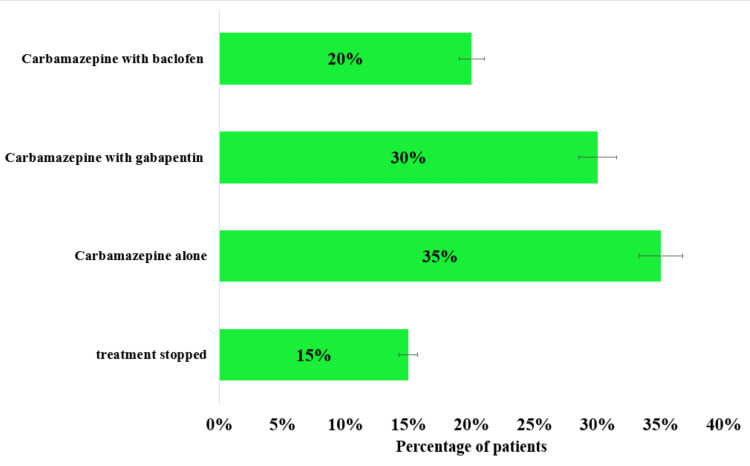
The distribution of patients based on their maintenance therapy outcomes for trigeminal neuralgia patients, including those who stopped treatment, continued with carbamazepine alone, carbamazepine with gabapentin, and carbamazepine with baclofen. The graph shows that 45 patients (15%) discontinued treatment, while 105 patients (35%) carbamazepine alone, 90 patients (30%) with carbamazepine and gabapentin, and 60 patients (20%) with carbamazepine and baclofen.

The results of the maintenance therapy for TN showed that 45 (15%) of patients discontinued treatment due to intolerable side effects or inadequate pain control. Common reasons for discontinuation included dizziness, fatigue, and adverse effects that impacted daily life, with some patients opting for surgical interventions instead. While 105 (35%) of patients successfully maintained pain control using carbamazepine alone, with dosages ranging from 200 to 800 mg per day. These patients generally experienced sustained relief, though some required periodic dose adjustments. A further 90 (30%) of patients continued on combination therapy with carbamazepine and gabapentin, which proved to be the most effective in reducing pain and minimizing side effects. Gabapentin doses ranged from 300 to 1,800 mg per day, allowing for lower doses of carbamazepine and fewer episodes of breakthrough pain. Finally, 60 (20%) of patients were maintained on a combination of carbamazepine and baclofen, particularly benefiting those with muscle spasms. Baclofen doses varied between 30 mg per day, providing adequate relief where carbamazepine alone was insufficient. Overall, combination therapies showed better long-term outcomes, while a small percentage of patients required alternative treatments due to poor response or side effects from pharmacological therapy (Figure 5).

In this study, dosage adjustments were made based on patient response. Among those on carbamazepine alone, 41 (30%) of patients required increased doses for better pain control, while 27 (20%) of patients had their dosage reduced due to side effects like dizziness, fatigue, etc. For patients on carbamazepine with gabapentin, 29 (35%) of patients had their gabapentin dose increased, and 13 (15%) of patients needed a reduction due to side effects like drowsiness and cognitive impairment. In the carbamazepine and baclofen group, 20 (25%) of patients required higher baclofen doses, while eight (10%) of patients had their dosage lowered to mitigate side effects such as muscle weakness and sedation. The study compares various pharmacological treatments for TN, revealing small but clinically significant differences in pain control. Even slight pain reductions can significantly enhance patients' quality of life. These differences may also improve adherence to therapy and reduce the need for invasive treatments, making them important considerations for personalized treatment decisions in clinical practice.

**Figure 6:**
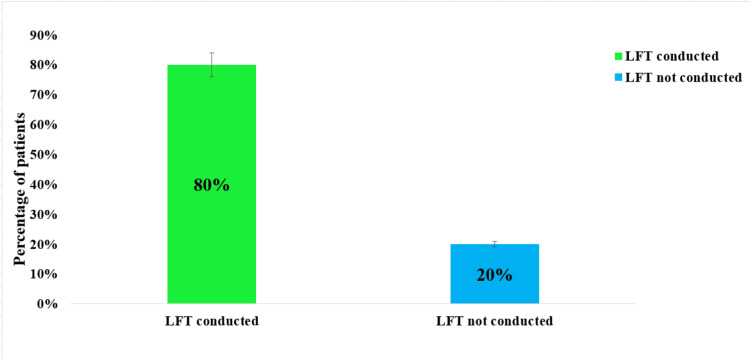
The percentage of patients who underwent liver function tests compared to those who did not. This graph shows the percentage of patients who had liver function tests (LFTs) conducted during the study; 80% of patients underwent LFTs, as represented by the green bar, while 20% of patients did not have LFTs performed. The large proportion of LFTs conducted highlights the importance of monitoring liver function in patients, likely due to the potential hepatotoxic effects of medications used for TN treatment, such as carbamazepine and gabapentin. The error bars suggest minimal variation in these percentages.

LFT results are vital for monitoring the safety of medications like carbamazepine and gabapentin, enabling early detection of hepatotoxicity and timely treatment adjustments for safer long-term use in TN patients. In this study, LFTs were conducted on 240 (80%) of the patients to assess the impact of treatment, particularly the use of carbamazepine, on liver enzymes and renal function. The results revealed that 60 (25%) of the patients had elevated levels of alanine aminotransferase (ALT) (56 IU/L) and aspartate aminotransferase (AST) (42 IU/L), which are key indicators of liver function. Additionally, 24 (10%) of patients showed elevated bilirubin levels (1.8 mg/dl), further indicating hepatic involvement. These findings underscore the necessity for routine LFT monitoring in patients undergoing treatment with medications like carbamazepine to prevent potential hepatotoxicity. The remaining 60 (20%) of the patients did not conduct LFTs in this study. The observed changes in liver enzyme levels were found to be statistically significant (p = 0.04) (Figure 6).

## Discussion

TN is a chronic pain condition marked by sudden, severe, electric shock-like pain in the face, most commonly affecting individuals in their fifth to sixth decade of life. [16]. Symptomatic TN is more common in younger individuals [17]. Our study also observed a similar trend, with the peak onset age occurring between the 50s and 60s. TN shows a gender preference, with a higher percentage of females compared with males. According to the literature, the female-to-male ratio is 5:3 [18]. Zakrzewska reported that after adjusting for the higher number of elderly adult women in the group, the gender distribution was equal [19]. Gender involved in TN showed that males have a higher rate than females [20]. Some studies indicate that the right side is affected more frequently than the left, with a 2:1 ratio, potentially due to the narrower foramen rotundum and ovale on the right side [21]. Previous research has shown that one-third of patients experience neuropathic pain in both the V2 and V3 divisions of the trigeminal nerve. However, our study revealed that the maxillary nerve (V2) is more commonly affected by TN, with an occurrence rate of 53.3%, compared to the mandibular nerve (V3), with an occurrence rate of 47%. Furthermore, some studies found that females were more frequently affected than males [22]. In our study, females develop TN (53%) than males (46%). 

The diagnostic criteria of TN rely on clinical observations, and the primary approach to management is generally non-surgical [23]. If patients do not respond well to medical treatment or are unable to tolerate the medications, a range of peripheral and central neurosurgical procedures may be considered as alternative options [24]. In this study, pain was controlled using carbamazepine drug alone and in combination with carbamazepine like gabapentin or baclofen. Carbamazepine is a gold-standard drug for the medical management of TN patients and has a well-documented track record of effectiveness in reducing or eliminating the sharp, stabbing pain associated with TN. It works by stabilizing hyperactive nerve membranes and blocking abnormal nerve impulses, which are the cause of TN pain [25]. In cases where carbamazepine is ineffective, or its dosage is restricted due to side effects, it can be combined with an AED or other antiepileptic [26]. Among the 50 patients treated with carbamazepine, 62% reported significant pain relief. However, during the follow-up period, 10% of these patients experienced one acute flare-up, requiring adjustments to their medication regimen [27]. 

Our findings indicate that carbamazepine continues to be a key component in the initial management of TN, providing pain relief for 72% of patients when used alone. However, the fact that 28% of patients did not achieve adequate pain relief emphasizes the necessity for alternative or adjunct therapies for a significant portion of the patient population. The study highlighted the importance of maintenance therapy in managing TN in the long term. Notably, the combination of carbamazepine with gabapentin showed the highest effectiveness (80%). Moreover, the discontinuation rate of 20% in patients who stopped treatment. Carbamazepine is combined with gabapentin or baclofen in patients who do not achieve adequate pain control with carbamazepine alone and experience intolerable side effects. Gabapentin enhances pain relief by targeting different pain pathways, while baclofen helps alleviate muscle spasms, offering a more comprehensive approach to managing TN symptoms [28, 29]. 

Blood investigations played a critical role in this study, particularly considering the chronic nature of the medication regimens required for TN management. The high rates of testing for liver and kidney functions (80%) align with the necessity to monitor for adverse effects of drugs like carbamazepine, which can cause hepatic and renal impairment. The comprehensive approach to monitoring underscores the need for vigilance in managing TN patients, particularly those on polypharmacy regimens.

In this study, dosage determination for pharmacological treatments, such as carbamazepine, was based on standard clinical guidelines and adjusted according to each patient’s response and tolerance. Initial doses were started at the lowest effective level and gradually increased until pain control was achieved or side effects became intolerable. Monitoring protocols included regular clinical follow-ups, where pain levels were assessed using the VAS, and LFTs were conducted periodically to detect potential hepatotoxicity, especially with long-term use of anticonvulsants. Side effects, such as dizziness, drowsiness, or liver enzyme elevation, were managed by either adjusting the dosage or switching to alternative medications like gabapentin or baclofen. If necessary, medications were tapered off or discontinued, and patients were closely monitored to ensure both safety and efficacy throughout the treatment period.

Regarding the medical management strategies for TN, our findings largely align with existing literature, confirming the effectiveness of pharmacological treatments such as carbamazepine and gabapentin as first-line therapies. However, our study uniquely highlights the role of adjunctive treatments like baclofen in patients who are refractory to standard therapies, which has been less emphasized in previous research. The limitations of this study, including its retrospective and institutional focus, may introduce selection bias and limit generalizability. Nevertheless, the data provide valuable insights into treatment patterns and patient outcomes. Strengths include the relatively large sample size and long-term follow-up, offering a comprehensive view of treatment efficacy over time. These findings underscore the importance of treatment approaches and suggest that further research should focus on prospective studies and exploring newer pharmacological agents or combination therapies. Clinically, the study supports the need for treatment plans, especially for patients unresponsive to first-line treatments, and it encourages the refinement of treatment algorithms for better patient outcomes. Long-term follow-up and research into treatment options, as well as medications, would improve the efficacy and safety of TN management regimens.

## Conclusions

The study highlights the effectiveness of medical treatments in managing TN, particularly the initial efficacy of carbamazepine and the enhanced outcomes associated with combination therapy. While these findings align with existing data that support carbamazepine as the first-line treatment, the study's results differ by emphasizing the superior pain relief provided by combination therapies, especially with gabapentin, which is less frequently highlighted in earlier research. This suggests that combination treatments may offer better long-term pain management than carbamazepine monotherapy, a departure from some of the established data that primarily focus on single-drug regimens. The study provides a framework for clinicians to develop comprehensive, individualized treatment plans that adapt to the evolving needs of TN patients. The findings advocate for a proactive approach to managing TN, with an emphasis on combination therapies to achieve sustained pain control and improve patient outcomes in the long term. Future research should continue to explore new drug combinations and personalized treatment strategies to further enhance the quality of life for those affected by TN.
